# Genomic differentiation among wild cyanophages despite widespread horizontal gene transfer

**DOI:** 10.1186/s12864-016-3286-x

**Published:** 2016-11-16

**Authors:** Ann C. Gregory, Sergei A. Solonenko, J. Cesar Ignacio-Espinoza, Kurt LaButti, Alex Copeland, Sebastian Sudek, Ashley Maitland, Lauren Chittick, Filipa dos Santos, Joshua S. Weitz, Alexandra Z. Worden, Tanja Woyke, Matthew B. Sullivan

**Affiliations:** 1 Department of Soil, Water and Environmental Science, University of Arizona, Tucson, AZ 85721 USA; 2 Department of Ecology and Evolutionary Biology, University of Arizona, Tucson, AZ 85721 USA; 3 Department of Molecular & Cellular Biology, University of Arizona, Tucson, AZ 85721 USA; 4 Department of Energy, Joint Genome Institute, Walnut Creek, CA 94598 USA; 5 Monterey Bay Aquarium Research Institute, Moss Landing, CA 95039 USA; 6 School of Biological Sciences, Georgia Institute of Technology, Atlanta, GA 30332 USA; 7 School of Physics, Georgia Institute of Technology, Atlanta, GA 30332 USA; 8 Integrated Microbial Biodiversity Program, Canadian Institute for Advanced Research, Toronto, M5G 1Z8 Canada; 9 Present Address: Departments of Microbiology, Ohio State University, Columbus, OH 43210 USA; 10 Present Address: Department of Evolution, Ecology, and Organismal Biology, Ohio State University, Columbus, OH 43210 USA; 11 Present Address: Department of Biological Sciences, University of Southern California, Los Angeles, CA 90089 USA; 12 Present Address: Department of Civil, Environmental and Geodetic Engineering, Ohio State University, Columbus, OH 43210 USA

**Keywords:** Bacteriophage, Phage, Cyanophage, Virus, Evolution, Species, Double-stranded DNA

## Abstract

**Background:**

Genetic recombination is a driving force in genome evolution. Among viruses it has a dual role. For genomes with higher fitness, it maintains genome integrity in the face of high mutation rates. Conversely, for genomes with lower fitness, it provides immediate access to sequence space that cannot be reached by mutation alone. Understanding how recombination impacts the cohesion and dissolution of individual whole genomes within viral sequence space is poorly understood across double-stranded DNA bacteriophages (a.k.a phages) due to the challenges of obtaining appropriately scaled genomic datasets.

**Results:**

Here we explore the role of recombination in both maintaining and differentiating whole genomes of 142 wild double-stranded DNA marine cyanophages. Phylogenomic analysis across the 51 core genes revealed ten lineages, six of which were well represented. These phylogenomic lineages represent discrete genotypic populations based on comparisons of intra- and inter- lineage shared gene content, genome-wide average nucleotide identity, as well as detected gaps in the distribution of pairwise differences between genomes. McDonald-Kreitman selection tests identified putative niche-differentiating genes under positive selection that differed across the six well-represented genotypic populations and that may have driven initial divergence. Concurrent with patterns of recombination of discrete populations, recombination analyses of both genic and intergenic regions largely revealed decreased genetic exchange across individual genomes between relative to within populations.

**Conclusions:**

These findings suggest that discrete double-stranded DNA marine cyanophage populations occur in nature and are maintained by patterns of recombination akin to those observed in bacteria, archaea and in sexual eukaryotes.

**Electronic supplementary material:**

The online version of this article (doi:10.1186/s12864-016-3286-x) contains supplementary material, which is available to authorized users.

## Background

Recombination can occur between closely and distantly related double-stranded DNA (dsDNA) phage genomes [1, 2]. The relative rate at which each of these types of recombination occurs can play an important role in determining if and how genomes move across sequence space. When an individual gains a genomic fitness advantage, maintaining its location in sequence space is essential for keeping the fitness advantage [3]. Recombination between closely related genomes can suspend the genome in sequence space by repairing slightly deleterious mutations, thereby preserving the fitness of the genome [4–6]. Individuals whose genomes are less fit can move across sequence space in larger steps via recombination, allowing them access areas of sequence space with higher fitness that cannot be reached by mutation alone [7]. Higher rates of close relative to distant recombination lead to and maintain the formation of discrete genotypes in sequence space. In contrast, the reverse can drive the formation of genomic continuums in sequence space [1, 8].

While genetic exchange plays an important role in maintaining and moving genomes in sequence space, high mutation rates were historically thought to overpower the impact of recombination in viral evolution resulting in the formation of quasispecies, mutant spectra of diverse genomic continuums [9]. This concept was built upon observations of RNA viruses, whose mutation rates mostly range from 10^−2^ to 10^−5^ mutations per nucleotide per generation [10]. In contrast, dsDNA viruses have mutation rates that fall consistently between 10^−7^ and 10^−8^ mutations per nucleotide per generation indicating that patterns of recombination may be strong enough to counteract mutation and lead to discrete population structure [10]. Recent work on dsDNA phages has consistently revealed discrete structure in wild populations [2, 11, 12]. Nonetheless, it remains unclear as to whether dsDNA phage populations in nature are discretely structured [11], or part of a continuum that is, as yet, insufficiently sampled [1, 8, 12].

Key to assessing dsDNA phage population structure is knowledge of the relative levels of close and distant genomic recombination. Despite analyses of recombination events across a subset of highly conserved genes [2], little work has been done to look at recombination across whole genomes. There are many lines of evidence that suggest recombination analyses across naturally coexisting whole genomes are necessary for accurately assessing the relative levels of close and distant recombination. First, recombination rates vary across genomes resulting in regions of high and low gene flow [13]. Further, many known viral recombination breakpoints occur outside or on the peripheries of genes [7, 14]. Lastly, only phages that coexist in the same environment and are able to infect the same host bacteria are able to undergo recombination. Thus, recombination analyses should be limited to whole genomes of individuals that can and could have potentially recombined.

Here we sought to establish and analyze a large-scale cyanophage genomic dataset to better understand the evolution of dsDNA phage genomes. Specifically, we characterized the structure and relationship of 142 closely-related cyanophage genomes isolated using a single host and only two source water samples. We assessed the impact of the relative rates of close and distant recombination in governing the emergence of discrete dsDNA cyanophage populations.

## Results and discussion

In order to investigate patterns of recombination across dsDNA phage populations, we chose to study wild dsDNA cyanophage communities. Cyanophages are abundant and ubiquitous in the global surface oceans [15, 16]. Further, many cyanobacterial hosts are already in culture [17]. These factors increased our chances of isolating multiple individual dsDNA phages from a single environment on a single host.

### Isolating dsDNA cyanophages to explore population structure

In total, 142 cyanophages were isolated using a single cyanobacterial host strain, *Synechococcus* WH7803; the strain which yielded the highest number of plaques of the Synechococcus strains available. While All 142 cyanophages were randomly isolated by picking all available well-separated plaques regardless of morphology, then triply plaque purified, grown to large volume, and whole genome sequenced to finished quality. Thus selection bias was limited only by plaque detection. To deeply evaluate isolate genomes in the environment, the 142 isolates were sourced from only two water samples such that 77 isolates were derived from a coastal water sample and 65 isolates were derived from an offshore, mesotrophic water sample. Both sites are part of the well-described California Cooperative Oceanic Fisheries Investigations Line 67 oceanographic transect, and represent ecologically distinct environments based on physical-chemical parameters (Additional file 1: Table S1) and proportions of host clades (Additional file 1: Figure S1), despite strong interconnecting currents [18]. All 142 genomes were sequenced and annotated and found to be myoviruses using transmission electron microscopy of representative isolates from each lineage (Additional file 2: Figure S2).

### Phylogenomic lineages represent discrete genotypic populations

**Fig. 1:**
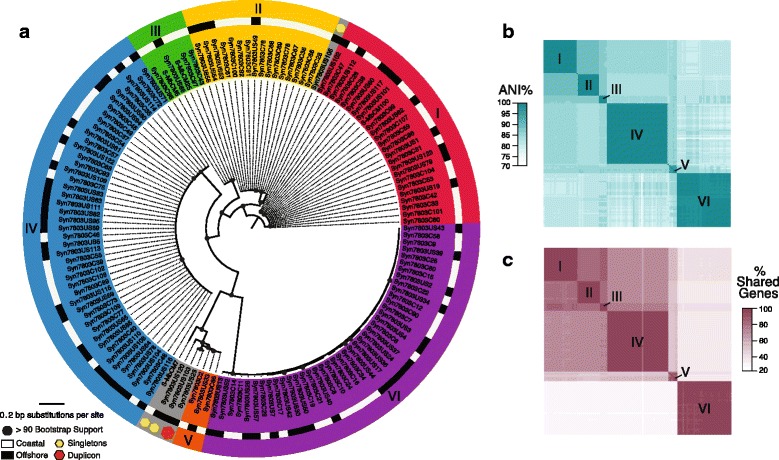
Phylogenomic analyses of 142 cyanophages. **a** Unrooted phylogenomic maximum likelihood tree of 51 concatenated core genes (see Additional file 2: Table S2) in 142 genome-sequenced isolates reveals 10 distinct cyanophage genomic lineages. Six lineages (designated I-VI) contain three or more representatives, while the remaining four are less well-represented and indicated by colored hexagons. Isolate origin (coastal = *white* or offshore = *black*) is designated in the outer ring. **b** Pairwise comparisons of average nucleotide identity (ANI) of shared genes between genomes in the well-represented lineages reveals six with ANI >98% that correspond to phylogenomic clusters I-VI. (**C**) The pairwise fraction of shared genes within clusters are high (>96%). Clustering of ANI and shared gene content are statistically not random (Additional file 2: Table S6) and correspond to bootstrapped phylogenomic lineages

Phylogenomic analyses of the 51 core genes (Additional file 2: Table S2) shared across all 142 viral genomes, revealed ten phylogenomic lineages of T4-like phage genomes (Fig. 1a), each well-supported by bootstrap analyses. Six lineages had at least three representatives enabling further within-lineage investigations. T4-like phage taxonomic classifications were confirmed by phylogenomic analyses using representative genomes from the literature (Additional file 2: Figure S3). The abundance of T4-like myoviruses in our isolate collections is consistent with the predominance of this virus type in (i) less culture-dependent viral-tagged metagenomes derived from these same coastal waters and using this same host [11], as well as (ii) global ocean surveys [19].

**Fig. 2:**
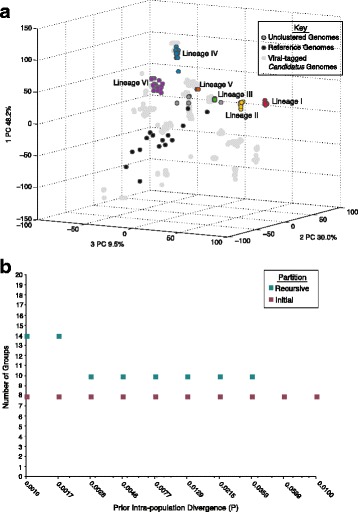
Alternative sequence-based clustering of genomes match phylogenomic lineages. **a** Principal component (PC) projection of the relationship between the 142 cyanophage genomes, previously sequences T4-like cyanophage genomes, and viral-tagged T4-like phages from the same coastal waters revealed tight clustering of genomes within the same phylogenetic lineage. Genomes were clustered based on ANI of genes within shared protein clusters. **b** Automatic barcode gap discovery method which sorts individuals in to the same population when divergence is smaller within than between also revealed identical clustering to the phylogenomic lineages

To determine if the phylogenomic lineages represented discrete genotypic populations, we looked at shared gene content, average nucleotide identity (ANI) of the shared genes, and gaps in the distribution of pairwise differences between genomes. Within a phylogenetic lineage, most (average >96%) genes were shared between genomes (Fig. 1b). Average nucleotide identity of across the 51 core genes was an average of >99% across the lineages (Fig. 1c) within and 80–89% between lineages (Additional file 2: Table S3). Further, adding the 142 genomes to the aforementioned viral-tagged metagenomic sequences we performed *in silico* sizing and positioning to arrange the viral-tagged sequences and genomes into sequence space using ANI (Fig. 2a). We found that the 142 genomes clustered tightly in sequence space with an average of >99% ANI across clusters, which is consistent with previous findings from viral-tagged metagenomes obtained from the same host [11]. These shared gene content percentages and ANI values are also relatively consistent with commonly observed thresholds in prokaryotes, which are commonly used to designate species cut-offs [20, 21].

As a separate metric for delineating separable populations, we next used the automatic barcode gap discovery method [22] to explore the distribution of pairwise differences between the 51 concatenated core genes. The method works by establishing the range of intra-population divergence values, then detecting gaps via an iterative process to partition the sequences into emergent discrete population boundaries. For our 142 cyanophage genomes, the number of populations predicted following recursion under multiple prior intra-population divergence values converged at ten populations (Fig. 2b) – all of which map identically to the phylogenomic lineages (Additional file 2: Figure S4). Taken together with the shared gene and ANI analyses, we interpret these phylogenomic lineages to represent discrete genotypic populations rather than artifacts of undersampling.

### Populations are distinct from each other

**Fig. 3:**
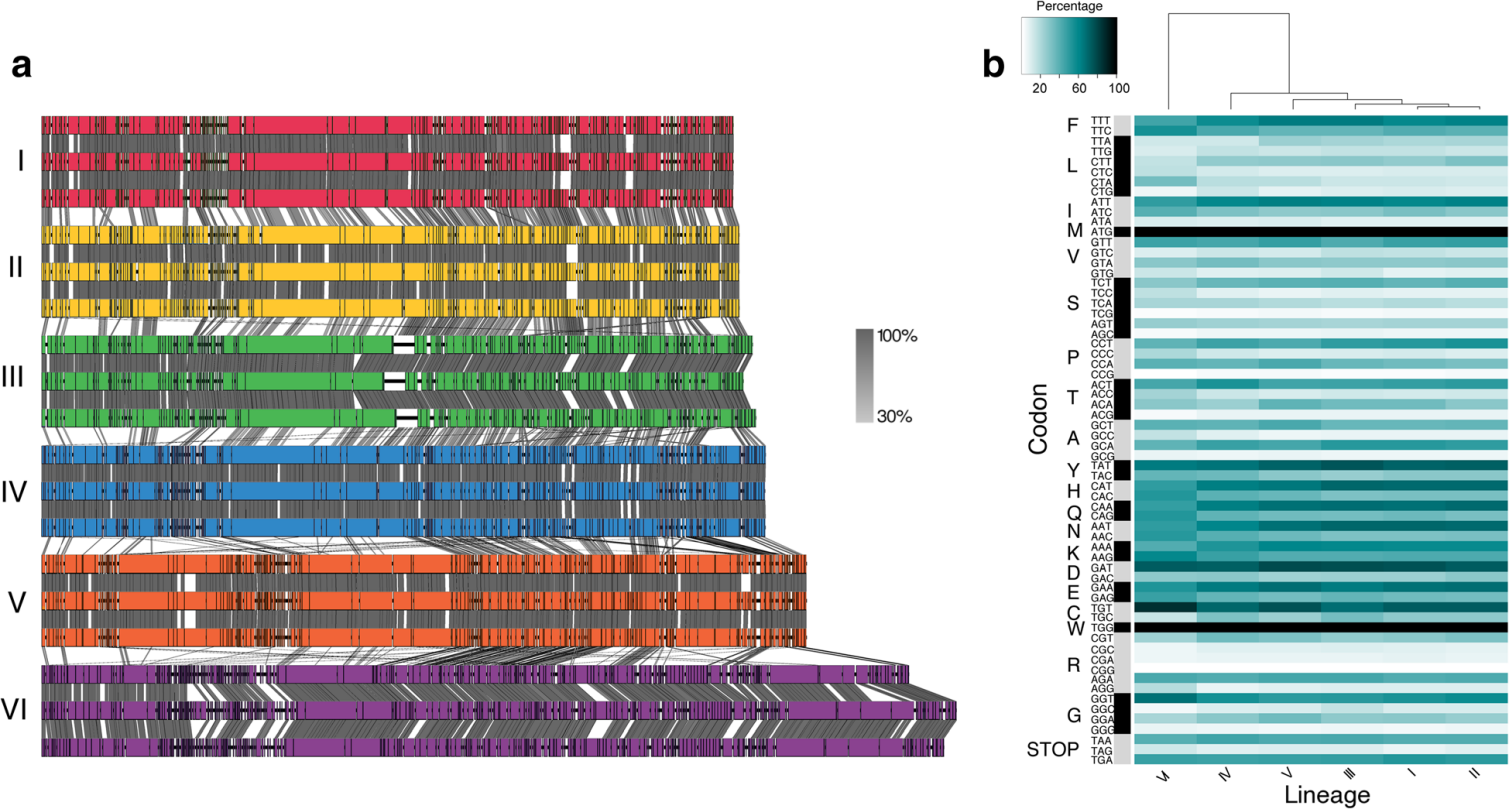
Comparative Genomics of lineages. **a** Synteny plot (blastn) within and between clusters shows high conservation of synteny within a cluster and between phylogenetically close lineages. Lineages V and VI shows an erosion of synteny. **b** Codon usage within each cluster reveals similar codon usage between lineages I-V with lineage VI as an outgroup

Of these ten populations, six are well-represented with one of these six (lineage VI) being the most divergent. Within a population and between closely related populations, there is a strong conservation of synteny (Fig. 3a). However, synteny breaks down between phylogenetically distant populations. Percent GC, genome length, and gene number are also highly conserved within a population (Table 1). One population, lineage IV, has one individual, Syn7803C55, whose genome, though complete based on our metric for a complete genome (refer to methods), is a statistical outlier (p-value < 0.05; Grubbs’ test) by genome length compared to the rest of the genomes in the lineage. Data from this genome was removed from the mean values of lineage IV. The mean percent GC ranged from 39.37 to 41.57% across lineages. Mean population genome length and gene number ranges from 171kbp to 221kbp and from 208 to 285 genes, respectively. Interestingly, population genome length and gene number increase with phylogenetic distance from lineage I to lineage VI suggesting that population differentiation events may correspond with increased access to novel gene pools.

**Table 1:** Genometrics for lineages

Lineage	Mean Genome Length (bp)	Mean GC%	Mean Gene Number
I	171,209 ± 330	39.37 ± 0.01	208 ± 2
II	171,588 ± 4,286	39.14 ± 0.02	216 ± 1
III	175,604 ± 855	39.10 ± 0.01	217 ± 4
IV	179,153 ± 85	40.27 ± 0.01	218 ± 1
V	189,419 ± 1	38.94 ± 0.00	215 ± 2
VI	221,263 ± 5,256	41.57 ± 0.04	285 ± 6

Table 1 Mean genome length, percent GC, and gene number across the populations reveals lineage VI as an distinct from the other lineages, with a much larger genomes size, higher percent GC, and higher gene number. For lineage IV, phage SynC55 was excluded from analyses.

Furthermore, one population (lineage VI) is significantly phylogenetically divergent (p-value < 0.01; two-tailed Mann-Whitney U-test) from the other five populations (Additional file 2: Table S5). It is the only population with a mean genome length greater than 200kbp and it has the highest percent GC and gene number. The high GC content may correlate with its different codon usage (Fig. 3b). It is the only population to have a different codon usage for both phenylalanine and leucine codons. This may suggest that while it can share a host with lineages I, II, III, IV, and V, it may be more efficient at infecting an alternative host and, thus, has an expanded gene pool paralleling the increase in genome size and gene number.

### Population boundaries are not caused by physical isolation

Because biogeography has large impacts on microbial diversity and abundances [23] as observed between our coastal and offshore sites (Additional file 1: Figure S1), we predicted that physical boundaries based on these host differences across the two sites might lead to genetic drift. Thus, we next evaluated whether genetic drift caused by physical boundaries across the two sites sampled may have a role in shaping the high genomic divergence between our genotypic populations. At least one member of all six well-represented populations could be found at both the coastal and offshore locations (Fig. 1a). All paired geographic populations (individuals of the same lineage at a given site), except those of lineage II, had low genetic differentiation (*F*_*ST*_ ≤ 0.0097; Additional file 3: Table S7), suggesting high gene flow between environmental sites. The cyanophage data presented here provide a first look at gene flow in ocean viruses, and are consistent with recent observations that “everything is everywhere, and the environment selects” derived from patterns observed in global ocean surveys using viral population genome fragments [24].

**Fig. 4:**
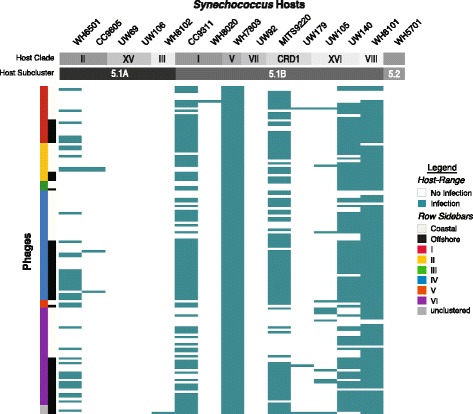
Host range analyses of 15 *Synechococcus* host strains against 138 cyanophage isolates. Four of the genome-sequenced isolates (S-MbCM6, 7, 25, and 100) were not examined. Cyanophage standard host ranges, i.e. infection or no infection host range, exhibit little correspondence with phylogenomic lineage or environmental origin with at least one member of each lineage sharing the ability to infect CC9311, WH7803, MITS9220, UW140, and WH8101, indicating a lack of physical boundaries between cyanophage populations. There is little correspondence between host range and phylogenomic lineages, even if the structure of the host range is unlikely to occur as a result of chance (see Additional file 3: Table S8)

Mechanistically, while gene flow in bacteria and archaea can occur from multiple sources, gene flow in viruses is restricted to times when two viruses co-infect the same host. Such “co-infection” need not be simultaneous, but it does require spatial proximity and shared host range. To quantify the role of this ‘host partitioning’ in driving physical boundaries between cyanophage lineages, we next conducted a large-scale host range analysis of 138 of the viral isolates against 15 diverse *Synechococcus* host strains. This revealed only minimal host range differences between lineages, as assessed via alternative clustering metrics (Fig. 4). This provides little support for host partitioning amongst our six cyanophage lineages, and contrasts observations in RNA phages where reproductive isolation appears driven by a decrease in shared host range [10]. We also assessed the infection efficiency of the phage isolates across the hosts via a quantitative host range (Additional file 3: Figure S4a). Again, we observed no significant difference in hierarchical clustering across lineages. However, we did observe a significant difference in infection efficiency between phages that were isolated from either the coastal or offshore sites in lineages I, II, IV and VI (Additional file 3: Figure S4b). This suggests that environmental conditions may alter the expression of infection genes, but not the presence or absence of genes or single nucleotide variants within genes themselves across these lineages.

Together these findings provided no support for the hypothesis that genetic drift caused by physical isolation – whether by spatial site or by host range – was a driving factor in the emergence of the observed cyanophage phylogenomic lineages.

### Population boundaries may be instigated by niche-differentiating genes

**Fig. 5:**
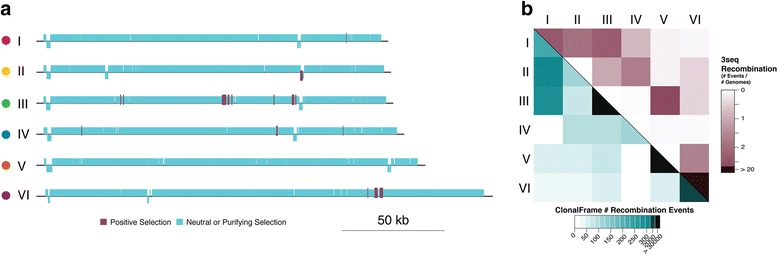
Selection and recombination results across lineages. **a** Non-polarized McDonald-Kreitman analyses reveal different selective signatures across phylogenomic lineages (Fisher’s exact tests, *p* < 0.05) and effect size cut-offs (phi coefficient ≥ 0.1). **b** The number of recombination events detected within and between lineages as inferred using coalescent (*turquoise*) and substitutions (*maroon*). Detailed gene annotations, *p*-values, and effect sizes for panel A are available in Additional file 3: Table S9

We next explored whether natural selection might facilitate the differentiation of the six dsDNA cyanophage populations. To this end, the genotypic populations were evaluated for differential selective pressures by non-polarized (i.e. pairwise) McDonald-Kreitman (MK) tests with low frequency slightly deleterious mutations removed from the analyses. These conditions are suggested to conservatively estimate positive selection in asexual organisms [25], but this remains a controversial area of the literature [26]. The MK tests detected selective signatures across all phylogenomic lineages (Fig. 5a) with p-values <0.05 and varying negative effect sizes (Additional file 3: Table S9). One population (lineage V; *n* = 3) had no detectable positive selection which could be due to low statistical power because of the low sample size. While positively selected genes varied across the populations, the number of signals was indistinguishable from a false positive rate across all populations. Thus, we cautiously interpret any MK signal here as it may either represent niche-driving selection or be an artifact.

If the positive selection signal is indeed biological, then it suggests niche differentiation across the populations, ranging from specialization in nucleotide synthesis (lineage III: *purL* and *dam*) to improving energy capture during infection (lineage VI: *hli03, psbA* and *psbD*). Admittedly, the small signal of positive selection on *psbA* and *psbD* in lineage VI may be artifactual as the effect size is small for these genes and the finding contrasts that of previous studies where these genes were under strong purifying selection [2]. Further, we evaluated the relative abundance of all genes putatively found under positive selection in the Global Ocean Viromes (GOV) populations [19]. The *gp14* neck protein was found to be under selection in lineage III and also found to be an abundant protein cluster specific to upwelling ocean regions and the Mediterranean Sea, indicating that it may be helpful in adapting to coastal regions. While the remaining genes were unannotated, one of them, MBARI_cyanophage_PC_42, is among the 100 most abundant protein clusters in the GOV populations, and present across 55 out of 91 of the epi- and meso-pelagic GOV samples. This suggests it may play a role in adapting to open ocean conditions. It should be re-noted that we should be cautious about of the selection results because the number of positive selection signals was indistinguishable from a false positive rate across all populations.

### Patterns of recombination maintain discreteness of genotypic populations

Leveraging an unprecedented genomic sequencing effort for dsDNA phages isolated on a single host at only two sites, we examined close (within) and distant (between) recombination in the 142 cyanophage genomes. The goal was to evaluate patterns within and between cyanophage genotypic populations to determine the extent to which niche differentiation may be reinforced by diminished gene flow. We predicted that recombination would play a strong role in our dataset because previous work, with eight conserved gene markers across 60 dsDNA cyanophages, revealed high levels of within-relative to between-population recombination [2].

To quantify gene flow, recombination events were identified across whole genomes using both substitution- and coalescent-based methods on datasets of core genes (genic) and core gene regions (intergenic and genic), respectively. Recombination was greater within- than between-population for three (III, V, and VI) populations by the coalescent method and two (I and VI) populations by the substitution-based method (Fig. 5b). This suggests that at least four of the six population boundaries were detectably maintained by recombination. Because the frequency of recombination events detected can be biased by sample size and nucleotide divergence, we evaluated such biases in our data. Sample size did not influence the frequency of recombination events detected, as the population with the lowest (V; *n* = 3) and second highest (VI; *n* = 42) number of representatives both had high within-population recombination. In contrast, nucleotide divergence likely did affect our ability to detect recombination, as recombination was not detected in lineage II and was only weakly detected in lineage IV, the populations with >99.8% nucleotide identity. Also, the highest number of recombination events was detected within the population with the most within-population divergence, lineage VI. These findings are consistent with prior suggestions that >5% nucleotide divergence is needed to detect recombination [27] and suggested that we are accurately measuring recombination between populations that all have >5% divergence (Table S3), but likely under-estimating recombination within our closely related populations [28]. Thus, we only felt confident in addressing cases where we observed higher between- than within population recombination.

Significantly, recombination breakpoints detected using RDP4, which integrates both substitution- and phylogenetic-based recombination detection methods, across the whole genome were identified outside of genic (i.e. intergenic) regions. Intergenic recombination breakpoints have been identified in both RNA [14] and ssDNA viruses [7]. While we only found intergenic recombination breakpoints of within lineage VI (~3%; Additional file 3: Table S10), we hypothesize this is because of the higher level of nucleotide divergence within lineage VI relative to the other lineages which allowed for the detection of multiple recombination events. This indicates that recombination can occur outside of genic regions and that analyses of full genomes rather than single genes is necessary to quantify the total relative rates of *inter-* versus *intra-* population recombination.

Overall, it appears that at least four of the six populations show patterns of gene flow that help maintain, rather than ameliorate, discrete population structure, and the remaining two of six populations appear too closely related to accurately quantify gene flow.

In contrast to our cyanophage findings, analyses in mycobacteriophages, where 1,540 genomes are available (http://phagesdb.org, 13 July 2016), have suggested that dsDNA phage sequence space represents a continuum that merely remains undersampled. Previous analyses of these genomes, mostly *Siphoviridae* and isolated using a single host, revealed genomic “clusters”, but the authors also infer rampant mosaicism or horizontal gene flow (i.e. recombination) events [12, 29–31] thought to evidence genomic continuity in sequence space. Notably, however, mycobacteriophage gene flow events were inferred from shared protein lineages, “phamilies” [12], defined by relatively low sequence identity (>32.5% amino acid identity [32]). While such permissive cut-offs are invaluable for comparative phage genomics, they measure evolutionary events much deeper than those relevant for speciation. Instead, to measure recombination events relevant for studying speciation, the inferences should be made using substitution, coalescent, or phylogenetic methods. Unfortunately, these cannot be used here due to vast undersampling at a single site, as only 1 or at most 2 phages were isolated per site. Thus, more intensive, per-site mycobacteriophage sampling are needed to explore homologous recombination events between “clusters” and better discriminate between a genomic continuum and discrete population structure.

Mechanistically, however, it is plausible that recombination could both maintain (as in the cyanophages) and amelioreate (as proposed for the mycobacteriophages) discrete population structure as these two phage groups may simply evolve differently. This is because the cyanophages and mycobacteriophages are predominantly lytic T4-like myoviruses and temperate *Siphoviridae,* respectively*.* The latter has been inferred via presence of an integration cassette [33], and confirmed experimentally for 4 of the mycobacteriophages (Tweety, Giles, BPs, and Halo [33]). Because temperate viruses may experience higher gene flow due to increased exposure to and recombination with foreign DNA resulting from the prolonged accessibility of their genomes within a cell in the prophage state [34] such as previously described in the mycobacteriophage Giles [35], it is possible that the lytic lifestyle of cyanophages could result in more population structure than the more rampant recombinogenic, temperate mycobacteriophages.

Recombination analyses on whole genomes of other wild, naturally coexisting dsDNA phage populations are necessary to determine if our results are broadly applicable to other dsDNA phages, such as the mycobacteriophages.

## Conclusions

While the role of recombination in maintaining discrete population structure is established and empirically defined among sexual eukaryotes [36], it remains controversial for bacteria, archaea [37, 38] and viruses [11, 12, 39]. Our findings suggest phylogenomic lineages in naturally occurring dsDNA cyanophages represent discrete genotypic populations. We hypothesize that discrete population boundaries are most likely initiated by sympatric niche differentiation rather than by allopatric isolation and that recombination maintains differentiated populations once established. Problematically, these latter conclusions are challenged by currently-insufficient methods to detect recombination and selection across closely-related genomes. Whether selection and recombination, rather than mutation and genetic drift, drive differentiation in other viruses remains an open question. This will undoubtedly vary along the spectrum of viral nucleic acid types and lifestyles – with minimal population structure among RNA and ssDNA viruses, and more population structure among dsDNA viruses. Together with microbial studies [40, 41], these cyanophage results suggest the potential for a unifying role of recombination in maintaining differentiation in populations across multiple scales – from sexual eukaryotes to bacteria and archaea to at least a subset of dsDNA phages.

## Methods

### Experimental methods

#### Host culturing techniques

*Synechococcus* strain WH7803 (SynWH7803) was grown at 20-22°C under a 14h:10h light-dark cycle at 15-18μE m^−2^s^−1^ in ‘SN’ medium [42], made from filtered (100kDa membrane, nominal molecular weight limit) and autoclaved water collected from surface Pacific Ocean (10 m depth, near White Point Park, San Pedro, California, USA; 33°40.51N, 118°13.76W). Liquid growth was followed using phycobiliprotein content as a proxy for biomass detected by fluorescence using an Appliskan plate reader (Thermo Electron, Vantaa, Finland; excitation wavelengths: 485 +/- 20 nm, emission wavelengths 590 +/- 40 nm).

#### Source waters

Water samples were collected from 10 m depth at stations H3 (36°44.34N, 122°01.20W; coastal) and 67-70 (36°07.56N, 123°29.46W; offshore) in Monterey Bay, CA, USA on the 1^st^ and 9^th^ of October 2009, respectively. Samples were immediately 0.22 μm filtered (Millipore Express Plus, Millipore, MA, US) and stored at 4°C in the dark in acid-washed polycarbonate bottles until further analysis. Nutrient measurements were conducted as previously described [43].

#### Phage isolation, imaging, DNA extraction and sequencing

Exponentially growing axenic *Synechococcus sp.* WH7803 cultures were transferred 1:5 by volume to fresh media and inoculated with 5 ml of either 0.22 μm filtered coastal or offshore seawater. Inoculations were serially diluted and plated using ‘SN’ media solidified with 0.05% low melting point agarose (Fisher, MA, US). Individual plaques, which appeared after 7 - 35 days, were collected in agarose plugs using sterile pipettes tips. Three rounds of plating were used to ensure well separated cyanophage plaques. Cyanophage isolates were grown in liquid culture on SynWH7803 to create lysate stock and stored at 4°C in the dark. Lysate stocks were used for experiments for up to six months and then regrown to minimize impact from viral decay. A randomly chosen isolate from each of the 10 cyanophage populations was imaged by transmission electron microscopy as previously described [44]. For genomic sequencing, 50 ml of phage lysate was PEG precipitated [45] for DNA extraction using the Wizard Genomic DNA Purification Kit (Promega, Fitchburg, WI). DNA was sequenced at the DOE Joint Genome Institute with 2x150bp Illumina MiSeq technology (Illumina, San Diego, CA) using a shotgun library prepared from plate-based DNA library preparation on the PerkinElmer Sciclone NGS robotic liquid handling system using Kapa Biosystems’ library preparation kit.

#### Quantitative host range (qHR) analysis

Viral lysates were serially diluted (10^0^, 10^−2^, 10^−4^, 10^−6^ and 10^−8^) in ‘SN’ media and tested on fifteen genetically diverse exponentially growing *Synechococcus* strains [17] (refer to Fig. 4) using plaque assays. Plaque formation was monitored for a total of 21 days with counts on day 7, 14 and 21. Plaque forming units (PFU) were determined based on the highest plaque count. The qHR analyses were conducted over the course of a year during which two different sets of viral lysate stock were used. To allow for comparison of viruses tested from each set, all phages from each set were tested for infectivity on SynWH7803 to enable normalization between the two different sets and also infectivity between the strains.

#### *Synechococcus* host 16S rRNA gene sequencing from field samples

DNA samples were extracted using a modification of protocols in the QIAGEN DNeasy kit [46]. Barcoded V1-V2 16S amplicons were amplified as described previously [47] and sequenced at the University of Arizona Genetics Core (Tucson, Arizona, USA) using 454 Titanium chemistry to produce >19,500 amplicons per sample.

### Bioinformatic analyses

#### Isolate genome assembly

Each fastq file processed with an in-house pipeline which filtered the read data for Illumina artifacts and human sequences using duk with options “-k 22 -s 1 -c 1” (http://duk.sourceforge.net/), kmer normalized to a target of 15X coverage with kmernorm with options “-k 21 -t 15 -c 2” (https://sourceforge.net/projects/kmernorm/), quality trimmed with FASTQX-toolkit fastqTrimmer version 0.0.13 (https://github.com/agordon/fastx_toolkit) with options “-b 5 -a 5 -l 45 -n 2 -p”, and assembled the filtered, normalized, and quality trimmed read data with VelvetOptimiser version 2.1.7 (https://github.com/tseemann/VelvetOptimiser), employing Velvet version 1.1.04 [48], with the following options; ”—v --s 51 --e 71 --i 4 --t 1 --o “-ins_length 250 -min_contig_lgth 500”. The resulting assembly was used to simulate 28X coverage of a long mate-pair library with insert 3000 +/- 270 bp using wgsim (https://github.com/lh3/wgsim) with options “-e 0 -1 100 -2 100 -r 0 -R 0 -X 0 ”. 25X of the simulated long-mate pair data was then coassembled with 125X coverage of the original QC filtered Illumina library with AllPathsLG release version R41043 [49]. Genomes can be found in GenBank under the accession numbers KJ019026- KJ019131, KJ019134- KJ019165, JN371768, and KF156338-40.

#### Genome protein clustering

Open reading frames (ORFs) were predicted using Prodigal [50]. A blastall of all ORFs against all ORFs was performed. Blast results were filtered for homologous genes [51]. Homology was defined as sequence similarity over 40% covering at least 60% of the length of the shortest gene. Using the blastall, ORFs were clustered with a granularity of 2 in MCL [52] using their workflow protocol for clustering similarity graphs encoded in blast results (http://micans.org/mcl/). In total 699 protein clusters were predicted, of which 51 were found across all 142 genomes; a sequence database of these 699 protein clusters is public (https://bitbucket.org/MAVERICLab/populationgenomics).

#### Detection of core genomic regions

Mugsy [53] was used to align locally collinear blocks of the within and between specific phage genomes based on phylogenomic and ANI lineage predictions. Variable regions from the alignment were stripped to leave only core alignment blocks greater than 500bp.

#### Phylogenomic analyses

For the nucleotide based tree on the 142 phage isolates, the 51 core gene clusters (see Table S1 for list) were aligned using the MAFFT [54] ^‘^–auto’ setting and analyzed for common T4-phage self-splicing introns. Briefly, where gene clusters copies were proximal to each other, the two sequences were analysed to see if they represented two different regions of the same gene. Where true, it indicated the presence of an intron, which was removed using a custom Perl script (remove_introns.pl; https://bitbucket.org/MAVERICLab/populationgenomics). Alignments were trimmed using GBLOCKS [55] with minimum number of sequences n × 0.5 + 1, maximum number of non-conserved columns 50, and minimum length of a block 5. Gene cluster alignments were then concatenated and the best-fit nucleotide substitution model was determined using PAUP* [56] and Modeltest 3.7 [57]. Phylogenetic reconstruction was conducted using RAxML [58] using the GTR substitution model and a gamma distribution in four discrete categories and a set of invariable sites (GTR + Γ + I).

For the protein tree, sequences were gathered from a selection of published marine and non-marine T4-like phage and combined with 142 cyanophage isolate genomes sequenced here. Sequences from unique gene clusters (*n* = 27) previously identified as “core genes” for T4-like phages [51] were processed as described for the nucleotide tree to remove introns, then analyzed. The best-fit amino acid substitution model was determined using prottest3 [59], and phylogenetic reconstruction was conducted using RAxML [58] with a WAG substitution model and a gamma distribution in four discrete categories and a set of invariable sites (WAG + Γ + I).

#### Shared genes, ANI, host range analyses

The percentage of pairwise genomic shared genes were determined based on the number of shared predicted protein clusters divided by the total number of protein clusters plus singletons within the genome. Blastn percent identities between all pairwise shared genes were averaged to calculate the average nucleotide identity (ANI). Host-range analyses were put in a matrix where a positive infection was given the value ‘1’ and the absence of infection was given the value ‘0.’ Heatmaps were created using the R program Heatmap3 [60] and 1000 hierarchical clustering bootstraps for both the rows and columns were performed using the R program pvclust [61]. To ensure that the clustering was not a result of chance, the rows and columns were randomized using 1000 iterations in the R program picante [62]. Pvclust hierarchical clustering of the original and randomized matrices were compared using the R program fpc [63] to obtain the level of clustering similarities (corrected Rand index and Malia’s VI).

#### Whole genome comparisons with previously published viral-tagged metagenomic sequences

To estimate the genomic relatedness of the newly defined lineages and the previously described populations (CGs *sensu* [11]) we adopted a similar approach with the variation that the original matrix was ANI not AAI. Briefly, genomic relatedness of the present lineages, previously obtained isolates and population genomes from the viral-tagging experiment [11] was compared using ANI; 2750622 ANI values were computed (17 available cyanophage genomes, 1500 viral tagged replicate genomes and 142 genomes from present study). ANIs was calculated only from conventionally defined pairs of homologous genes [51]. Homology was defined as sequence similarity over 40% covering at least 60% of the length of the shortest gene. The matrix of pairwise ANI genome comparisons (size: 1,659 × 1,659) was used in principal component analysis, values were first arcsin transformed. The first three components account for 87.7% of the variation.

#### Automatic Barcode Gap Discovery (ABGD)

ABGD [22] version Jul 2014 was downloaded on Oct 14, 2015. While there is no universal barcoding gene available for phages, the 51 core genes shared by all of the 142 sequenced cyanophages were chosen as the dataset appropriate for this DNA barcoding analysis of a closely related virus community. Core genes of T4-like cyanophages display a signal of shared evolutionary history, as well as reduced effects of horizontal gene transfer evident in the flexible genome [64], and address part of the need for a dataset for which the evolution within a species follows that of a single unstructured population.

The program was run using the default Jukes-Cantor distance and 1.5X barcode gap options, with the -a command line option to output trees and text descriptions of all ABGD partitions. Prior divergence distances from 0.001 substitutions per site to 0.1 substitutions per site were tested, spanning common 5% divergence cutoffs used for analysis of microbial barcoding data [20, 21].

#### Genometrics

Custom Perl scripts using the BioPerl Bio:: Seq module were used to calculate the means and standard deviations of genome length, gene number, and percent GC across lineages. The script genometrics.pl can be found on https://bitbucket.org/MAVERICLab/populationgenomics. T-tests, U-tests, and Grubbs’ tests were performed in R.

#### F_ST_

The fixation indices (F_ST_) were calculated between the core genomics regions of all coastal and offshore populations of each phage lineages using the standard definition equation: ${F}_{ST}\left({S}_1,{S}_2,G\right)=1-\frac{\pi_{within}}{\pi_{between}}=1-\frac{\left(\pi \left({S}_1,G\right)+\pi \left({S}_2,G\right)\right)/2}{\pi \left({S}_1,{S}_2,G\right)}.$

The BioPerl Bio:: PopGen:: Statistics module [65] was used to calculate the nucleotide diversity (π) input for F_ST_.

#### Recombination analyses

For the coalescent-based recombination detection, the Bayesian-based ClonalFrame [66] was used to measure the frequency of recombination within and between different phage lineages using the core genomic regions aligned using mugsy. ClonalFrame predicted clonal genealogy after 10,000 burn-in iterations and 10,000 iterations based on the convergence of genealogies of triplicate runs. Recombination was evaluated using the number of detected recombination events (R).

For the substitution-based recombination detection, 3seq [67] was used genes that were clustered together into protein clusters. 3seq was used rather than RDP4 [68] for this step due to the ability to do a high-throughput analyses using command line. The genes were aligned with the MAFFT [54] ‘-auto’ setting and self-splicing introns were removed as above. Recombination events detected within and between each lineage were divided by the number of viruses within a lineage and the sum of viruses between both lineages, respectively.

Identification of genic versus intergenic recombination breakpoints was performed on the breakpoints identified by RDP4 [68] across the core genomic regions. A blastn was performed on sequences extending 50bp preceding the loci location of the recombination breakpoint in the core genomic alignments against the full genomes of individuals within a lineage to identify the location of the breakpoint within full genomes. Whether the breakpoint resided within a gene or intergenic region was determined using the location of the breakpoint within the full genome.

#### McDonald-Kreitman (MK) selection tests

Non-polarized MK tests were calculated on all shared genes between all phage lineages. It is important to note that performing MK tests between all phage lineages was merely to show that detectable positive selection has occurred at some point along the phylogeny at the locus. As previously stated, genes were aligned with the MAFFT [54] ‘-auto’ setting. Polymorphisms segregating in less than <15% of individuals within a lineage were removed from the analyses [69]. Custom Perl scripts (DetectSynandNonSyn.pl and MKselection.pl; https://bitbucket.org/MAVERICLab/populationgenomics) were used to detect the number of synonymous and nonsynonymous substitutions and run a two-tailed Fisher’s exact tests (*p* < 0.05) using the BioPerl Bio:: PopGen::Statistics module [65] to determine the value of the ratio of nonsynonymous to synonymous variation, respectively. The effect size of each positively selected gene, as measured by the phi coefficient, reveals relationship between fixed and polymorphic ratios of nonsynonymous to synonymous substitutions and polymorphisms, respectively. The phi coefficient was calculated using the following equation: $\varnothing =\frac{ad-bc}{\sqrt{efgh}}$ where *a* represents the number of synonymous substitutions per gene, *b* represents the number of synonymous polymorphisms per gene, *c* represents the number of nonsynonymous substitutions per gene, *d* represents the number of nonsynonymous polymorphisms per gene, *e* equals the sum of *a* and *b*, *f* equals the sum of *c* and *d*, *g* equals the sum of *a* and *c,* and *h* equals the sum of *b* and *d*. Lastly, a blastp of the genes that were found to be under positive selection was performed against the protein clusters in the GOV population dataset [19].

#### *Synechococcus* host 16S rRNA amplicon analyses

16S amplicon sequences were classified using the Phyloassigner pipeline as in [47] allowing initial identification of plastid and cyanobacterial sequences. These were then used in a second Phyloassigner run to assign them to a validated cyanobacterial 16S rRNA gene reference tree [18]. A total of 477 sequences from the coastal sample were assigned to *Synechococcus* clades. For 67-70, 1448 (2 Oct. 2009) and 4050 (10 Oct. 2009) amplicons were assigned to *Synechococcus*. The data is represented as the percentage of total *Synechococcus* sequences from each respective sample.

## Additional files

Additional file 1: Metadata. **Table S1.** Metadata from the coastal (H3) and mesotrophic (67-70) sites. Samples for the 16S rRNA amplicons were collected twice at 67-70, 8 days apart (with the 10 Oct. sample being done on the same day as the viral sample). The mesotrophic station is often subject to upwelling. **Figure S1.** Sea surface temperatures (SST) of the region of the California Cooperative Oceanic Fisheries Investigations (CalCOFI) Line 67 ocean transect on 5 October 2009 (contoured from a single synoptic image, Aqua Modis, NOAA) with the locations of the nearshore (yellow, H3 - coastal) and offshore (red, 67-70 - offshore mesotrophic) stations marked with stars. Gene marker (16S rRNA gene amplicons) analyses using the reference alignments of ref. Sudek *et al.*, 2015 revealed different *Synechococcus* communities at the two sites (for additional details on sampling details see Additional file 2: Table S1). The *Synechococcus* community was analyzed twice at 67-70, one day after the coastal sampling and on the same day as the viral 67-70 sample collection. Proportions of different clades varied in the 67-70 *Synechococcus* amplicon data but the same clades were present on both dates. (DOC 721 kb)
Additional file 2: Lineage information. **Figure S2.** TEM images of phage isolates from cyanophage (**A**) lineage I, (**B**) lineage II, (**C**) lineage III, (**D**) lineage IV, (**E**) lineage V, (**F**) lineage VI, and (**G-J**) singleton and duplicon populations confirms myovirus morphology. **Table S2.** List of 51 core protein clusters shared across all six phylogenetic lineages. **Fig. S3.** Unrooted phylogenomic maximum likelihood tree of 27 concatenated protein sequences shared across published marine and non-marine T4-like phage genomes and the 142 cyanophage isolate genomes sequenced here. For simplicity, cyanophage isolate names in this tree were shortened from Syn7803* to just *. These analyses show that the 10 cyanophage populations observed here share similar evolutionary histories with other T4-like phages. **Table S3.** Average ANI of the 51 core genes within and between lineages. **Table S4.** AGDB groupings correspond with the phylogenetic lineages. **Table S5.** Average phylogenetic distances within and between lineages. **Table S6.** Corrected Rand Indices and Malia’s VI values to compare the row and column hierarchical clustering between the original ANI and Shared Gene matrix and a randomized ANI and Shared Gene matrix, respectively. The hierarchical clusters were split into different number of clusters (5,10, 20 and 50) for the analyses. The analyses revealed low correspondence between clustering, indicating that the clustering we observe in the original matrices are not random. (DOC 4227 kb)
Additional file 3: Testing genetic drift, selection, and recombination. **Table S7.** F_ST_ values for genetic differentiation between coastal and offshore populations within each cyanophage lineage. The F_ST_ value for lineage II is most likely high due to the low number of representatives and lack of diversity among the offshore lineage II phages. Due to the low number of individuals isolated from the offshore site for some of the clusters, F_ST_ could not be calculated. **Figure S4. (A)** Quantitative host range analyses of 15 *Synechococcus* host strains against 138 cyanophage isolates testing the efficacy of infection. **(B)** Analysis of mean infectivity of coastal and upwelling phages in lineage I, II, IV, and VI reveal statistically different infectivity phenotypes at either site with T-test *p* <0.05 (*). Statistical significance was not assessed for lineages III and V due to low sample size nor on the original isolation host, WH7803 (†). **Table S8.** Corrected Rand Indices and Malia’s VI values to hierarchical clustering between the original host range matrix and a randomized host range matrix. The hierarchical clusters were split into different number of clusters (5,10, 20 and 50) for the analyses. The analyses revealed low correspondence between clustering, indicating that the clustering we observe in the original shared genes matrix is not random and that there is some correlation with a biological signal. **Table S9.** Fisher’s exact tests *p*-values and phi coefficients (for effect size) for genes found under positive selection in comparisons between phylogenomic lineages using the non-polarized McDonald-Kreitman test. Also, the table reflects the corresponding protein clusters in the GOV population dataset [19]. **Table S10.** Genic versus intergenic recombination breakpoints for each lineage. (DOC 250 kb)

## Abbreviations

ANI: Average nucleotide identity
GOV: Global oceans viromes
