# Supplementary material for: Genomic differentiation among wild cyanophages despite widespread horizontal gene transfer
Source: BMC Genomics. 2016 Nov 16;17:930. doi: 10.1186/s12864-016-3286-x (PMC5112629; doi:10.1186/s12864-016-3286-x)
Supplement: Additional file 1: — Metadata. Table S1. Metadata from the coastal (H3) and mesotrophic (67-70) sites. Samples for the 16S rRNA amplicons were collected twice at 67-70, 8 days apart (with the 10 Oct. sample being done on the same day as the viral sample). The mesotrophic station is often subject to upwelling. Figure S1. Sea surface temperatures (SST) of the region of the California Cooperative Oceanic Fisheries Investigations (CalCOFI) Line 67 ocean transect on 5 October 2009 (contoured from a single synoptic image, Aqua Modis, NOAA) with the locations of the nearshore (yellow, H3 - coastal) and offshore (red, 67-70 - offshore mesotrophic) stations marked with stars. Gene marker (16S rRNA gene amplicons) analyses using the reference alignments of ref. Sudek et al., 2015 revealed different Synechococcus communities at the two sites (for additional details on sampling details see Additional file 2: Table S1). The Synechococcus community was analyzed twice at 67-70, one day after the coastal sampling and on the same day as the viral 67-70 sample collection. Proportions of different clades varied in the 67-70 Synechococcus amplicon data but the same clades were present on both dates. (DOC 721 kb) [file 12864_2016_3286_MOESM1_ESM.doc]

**Additional file 1: Metadata**

**Table S1.** Metadata from the coastal (H3) and mesotrophic (67-70) sites. Samples for the 16S rRNA amplicons were collected twice at 67-70, 8 days apart (with the 10 Oct. sample being done on the same day as the viral sample). The mesotrophic station is often subject to upwelling.

**Fig. S1.** Sea surface temperatures (SST) of the region of the California Cooperative Oceanic Fisheries Investigations (CalCOFI) Line 67 ocean transect on 5 October 2009 (contoured from a single synoptic image, Aqua Modis, NOAA) with the locations of the nearshore (yellow, H3 - coastal) and offshore (red, 67-70 - offshore mesotrophic) stations marked with stars. Gene marker (16S rRNA gene amplicons) analyses using the reference alignments of ref. Sudek *et al.*, 2015 revealed different *Synechococcus* communities at the two sites (for additional details on sampling details see Additional file 2: Table S1). The *Synechococcus* community was analyzed twice at 67-70, one day after the coastal sampling and on the same day as the viral 67-70 sample collection. Proportions of different clades varied in the 67-70 *Synechococcus* amplicon data but the same clades were present on both dates.

**Table S1:**

| **Site** | **Sample Collected** | **Date** | **Location (Lat.;Lon.)** | **Depth (m)** | **T**  **(°C)** | **Salinity** | **PO_4_^3-^ (μM)** | **NO_3_ -(μM)** | **NH_4_^+^ (μM)** | **Chl*a* (mg m^-3^)** |
| --- | --- | --- | --- | --- | --- | --- | --- | --- | --- | --- |
| H3 (Coastal) | Viral & Bacterial | October 1, 2009 | 36°44.34N, 122°01.20W | 10 | 11.58 | 33.36 | 1.153 | 12.91 | 0.263 | 1.985 |
| 67-70 (Offshore) | Bacterial | October 2, 2009 | 36°07.56N, 123°29.46W | 10 | 16.43 | 33.19 | 0.320 | 0.40 | <0.010 | 0.496 |
| 67-70 (Offshore) | Viral & Bacterial | October 10, 2009 | 36°07.56N, 123°29.46W | 10 | 15.83 | 33.22 | 0.485 | 0.26 | 0.047 | 0.500 |

**Fig. S1:**
