# Supplementary material for: Genomic differentiation among wild cyanophages despite widespread horizontal gene transfer
Source: BMC Genomics. 2016 Nov 16;17:930. doi: 10.1186/s12864-016-3286-x (PMC5112629; doi:10.1186/s12864-016-3286-x)
Supplement: Additional file 2: — Lineage information. Figure S2. TEM images of phage isolates from cyanophage (A) lineage I, (B) lineage II, (C) lineage III, (D) lineage IV, (E) lineage V, (F) lineage VI, and (G-J) singleton and duplicon populations confirms myovirus morphology. Table S2. List of 51 core protein clusters shared across all six phylogenetic lineages. Fig. S3. Unrooted phylogenomic maximum likelihood tree of 27 concatenated protein sequences shared across published marine and non-marine T4-like phage genomes and the 142 cyanophage isolate genomes sequenced here. For simplicity, cyanophage isolate names in this tree were shortened from Syn7803* to just *. These analyses show that the 10 cyanophage populations observed here share similar evolutionary histories with other T4-like phages. Table S3. Average ANI of the 51 core genes within and between lineages. Table S4. AGDB groupings correspond with the phylogenetic lineages. Table S5. Average phylogenetic distances within and between lineages. Table S6. Corrected Rand Indices and Malia’s VI values to compare the row and column hierarchical clustering between the original ANI and Shared Gene matrix and a randomized ANI and Shared Gene matrix, respectively. The hierarchical clusters were split into different number of clusters (5,10, 20 and 50) for the analyses. The analyses revealed low correspondence between clustering, indicating that the clustering we observe in the original matrices are not random. (DOC 4227 kb) [file 12864_2016_3286_MOESM2_ESM.doc]

**Additional file 2: Lineage information**

**Fig. S2.** TEM images of phage isolates from cyanophage (**A**) lineage I, (**B**) lineage II, (**C**) lineage III, (**D**) lineage IV, (**E**) lineage V, (**F**) lineage VI, and (**G-J**) singleton and duplicon populations confirms myovirus morphology.

**Table S2.** List of 51 core protein clusters shared across all six phylogenetic lineages.

**Fig. S3.** Unrooted phylogenomic maximum likelihood tree of 27 concatenated protein sequences shared across published marine and non-marine T4-like phage genomes and the 142 cyanophage isolate genomes sequenced here. For simplicity, cyanophage isolate names in this tree were shortened from Syn7803* to just *. These analyses show that the 10 cyanophage populations observed here share similar evolutionary histories with other T4-like phages.

**Table S3.** Average ANI of the 51 core genes within and between lineages.

**Table S4.** AGDB groupings correspond with the phylogenetic lineages.

**Table S5.** Average phylogenetic distances within and between lineages.

**Table S6.** Corrected Rand Indices and Malia’s VI values to compare the row and column hierarchical clustering between the original ANI and Shared Gene matrix and a randomized ANI and Shared Gene matrix, respectively. The hierarchical clusters were split into different number of clusters (5,10, 20 and 50) for the analyses. The analyses revealed low correspondence between clustering, indicating that the clustering we observe in the original matrices are not random.

**Fig. S2:**

**
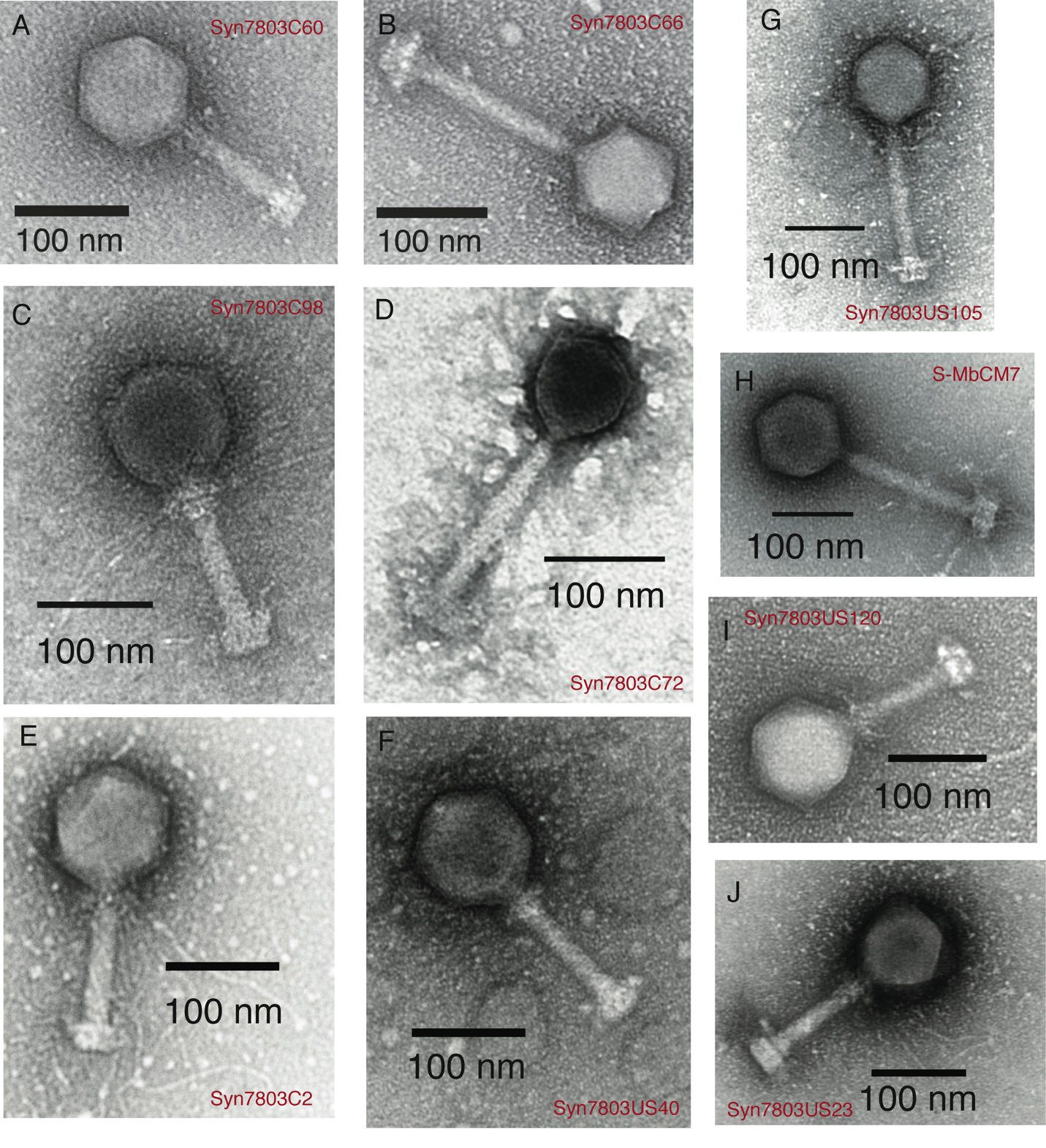
**

**Table S2:**

| **Protein Cluster** | **Annotation** |
| --- | --- |
| MBARI_Cyanophage_PC_1 | gp43 DNA polymerase |
| MBARI_Cyanophage_PC_10 | gp22 scaffold prohead core protein |
| MBARI_Cyanophage_PC_11 | CobS porphyrin biosynthetic protein |
| MBARI_Cyanophage_PC_12 | gp6 baseplate wedge protein |
| MBARI_Cyanophage_PC_14 | gpre46 combination endonuclease |
| MBARI_Cyanophage_PC_15 | UvsX RecA-like protein |
| MBARI_Cyanophage_PC_16 | hypothetical protein |
| MBARI_Cyanophage_PC_17 | cytitidyltransferase |
| MBARI_Cyanophage_PC_18 | ribonucleotide reductase subunit B |
| MBARI_Cyanophage_PC_19 | gp32 ssDNA binding protein |
| MBARI_Cyanophage_PC_20 | gp4 head completion protein |
| MBARI_Cyanophage_PC_21 | gp19 tail tube monomer protein |
| MBARI_Cyanophage_PC_22 | gp26 baseplate hub subunit |
| MBARI_Cyanophage_PC_23 | hypothetical protein |
| MBARI_Cyanophage_PC_24 | gp44 clamp loader subunit |
| MBARI_Cyanophage_PC_25 | gp25 base plate wedge subunit |
| MBARI_Cyanophage_PC_26 | gp13 neck protein |
| MBARI_Cyanophage_PC_27 | gp14 neck protein |
| MBARI_Cyanophage_PC_28 | gp15 proximal tail sheath protein |
| MBARI_Cyanophage_PC_29 | gp16 terminase DNA packaging protein |
| MBARI_Cyanophage_PC_3 | putative short tail fiber |
| MBARI_Cyanophage_PC_30 | hypothetical protein |
| MBARI_Cyanophage_PC_31 | PetE plastocyanine |
| MBARI_Cyanophage_PC_32 | gp17 terminase large subunit |
| MBARI_Cyanophage_PC_33 | gp3 head-proximal tip of tail protein |
| MBARI_Cyanophage_PC_34 | gp20 portal vertex protein |
| MBARI_Cyanophage_PC_35 | gp21 prohead core scaffold protein |
| MBARI_Cyanophage_PC_36 | head vertex gp24 precursor |
| MBARI_Cyanophage_PC_37 | hypothetical protein |
| MBARI_Cyanophage_PC_38 | UvsY |
| MBARI_Cyanophage_PC_39 | gp55 late transcription sigma factor |
| MBARI_Cyanophage_PC_40 | gp47 recombination endonuclease |
| MBARI_Cyanophage_PC_41 | peptidase |
| MBARI_Cyanophage_PC_42 | hypothetical protein |
| MBARI_Cyanophage_PC_43 | hypothetical protein |
| MBARI_Cyanophage_PC_44 | DNA adenine methylase Dam |
| MBARI_Cyanophage_PC_45 | translation repressor RegA |
| MBARI_Cyanophage_PC_46 | Hsp20 heat shock protein |
| MBARI_Cyanophage_PC_47 | gp41 DNA primase-helicase |
| MBARI_Cyanophage_PC_48 | MazG pyrophosphatase |
| MBARI_Cyanophage_PC_49 | hypothetical protein |
| MBARI_Cyanophage_PC_5 | NrdA ribonucleotide reductase subunit A |
| MBARI_Cyanophage_PC_50 | hypothetical protein |
| MBARI_Cyanophage_PC_51 | gp61 DNA primase subunit |
| MBARI_Cyanophage_PC_52 | TalC transaldolase-like protein |
| MBARI_Cyanophage_PC_53 | hypothetical protein |
| MBARI_Cyanophage_PC_54 | Hli03 high light inducible protein |
| MBARI_Cyanophage_PC_6 | gp18 tail sheath monomer protein |
| MBARI_Cyanophage_PC_7 | gp45 sliding clamp DNA polymerase |
| MBARI_Cyanophage_PC_8 | gp62 clamp loader subunit |
| MBARI_Cyanophage_PC_9 | UvsW RNA-DNA + DNA-DNA helicase |

**Fig. S3:**

**
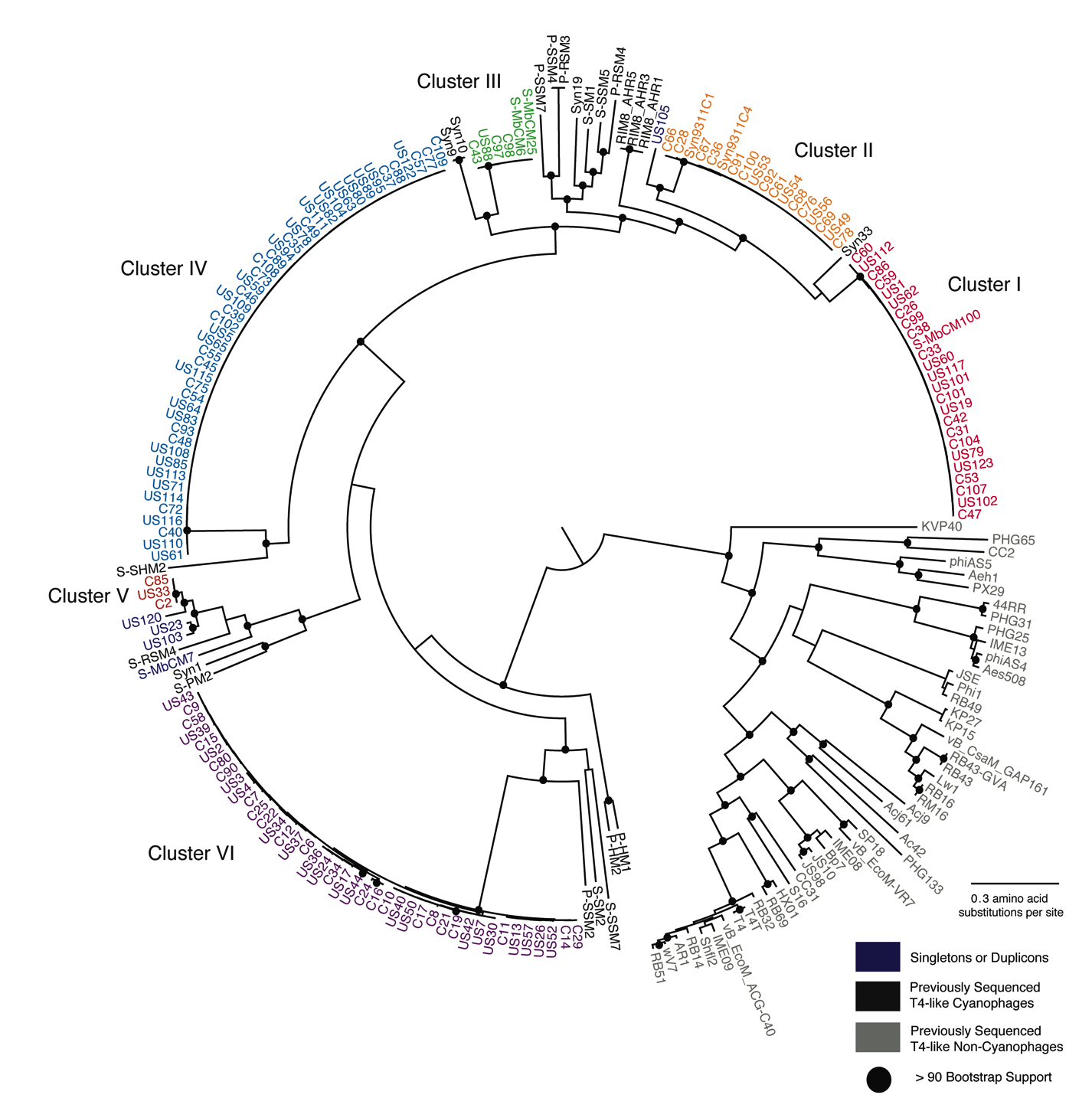
**

**Table S3:**

| **ANI** | **I** | **II** | **III** | **IV** | **V** | **VI** |
| --- | --- | --- | --- | --- | --- | --- |
| **I** | 99.63 |  |  |  |  |  |
| **II** | 89.12 | 99.86 |  |  |  |  |
| **III** | 86.83 | 87.71 | 99.69 |  |  |  |
| **IV** | 84.56 | 84.17 | 84.76 | 99.79 |  |  |
| **V** | 85.52 | 86.78 | 87.86 | 86.72 | 99.80 |  |
| **VI** | 81.12 | 80.67 | 83.00 | 83.71 | 84.33 | 98.23 |

Table S4:

| **ABDG Group** | **Lineage / Genome** |
| --- | --- |
| 1 | Lineage I |
| 2 | Lineage III |
| 3 | S-MbCM7 |
| 4 | Lineage II |
| 5 | Lineage IV |
| 6 | Lineage VI |
| 7 | Lineage V |
| 8 | Syn7803US105 |
| 9 | Syn7803US103 and Syn7803US23 |
| 10 | Syn7803US120 |

**Table S5:**

| **Phylogenetic Distances** | **I** | **II** | **III** | **IV** | **V** | **VI** |
| --- | --- | --- | --- | --- | --- | --- |
| **I** | 0.00350 |  |  |  |  |  |
| **II** | 0.30303 | 0.00129 |  |  |  |  |
| **III** | 0.45851 | 0.48526 | 0.00218 |  |  |  |
| **IV** | 0.76271 | 0.79578 | 0.73526 | 0.00156 |  |  |
| **V** | 1.32497 | 1.32035 | 1.12598 | 1.33179 | 0.00066 |  |
| **VI** | 1.63019 | 1.66117 | 1.59123 | 1.60790 | 1.59830 | 0.02120 |

**Table S6:**

| **Matrix** | **Row or Column (R or C) hierarchical clustering** | **Number of clusters compared** | **Corrected Rand Index** | **Malia’s VI** |
| --- | --- | --- | --- | --- |
| ANI matrix | R | 5 | 0.01285555 | 1.618112 |
|  | C | 5 | 0.002062033 | 1.653486 |
|  | R | 10 | 0.02757251 | 2.062771 |
|  | C | 10 | 0.001285499 | 2.094621 |
|  | R | 20 | -0.000938368 | 2.938377 |
|  | C | 20 | 0.02893305 | 3.050317 |
|  | R | 50 | 0.005733335 | 2.617428 |
|  | C | 50 | 0.006325598 | 2.615241 |
| Shared Genes matrix | R | 5 | -0.01267573 | 1.71286 |
|  | C | 5 | 0.007849774 | 1.528672 |
|  | R | 10 | -0.00761269 | 2.123239 |
|  | C | 10 | -0.002966576 | 2.334517 |
|  | R | 20 | 0.04993382 | 2.802539 |
|  | C | 20 | 0.01734365 | 2.935722 |
|  | R | 50 | -0.01710149 | 2.790352 |
|  | C | 50 | -0.01396406 | 2.79337 |
