# Supplementary material for: Genomic differentiation among wild cyanophages despite widespread horizontal gene transfer
Source: BMC Genomics. 2016 Nov 16;17:930. doi: 10.1186/s12864-016-3286-x (PMC5112629; doi:10.1186/s12864-016-3286-x)
Supplement: Additional file 3: — Testing genetic drift, selection, and recombination. Table S7. FST values for genetic differentiation between coastal and offshore populations within each cyanophage lineage. The FST value for lineage II is most likely high due to the low number of representatives and lack of diversity among the offshore lineage II phages. Due to the low number of individuals isolated from the offshore site for some of the clusters, FST could not be calculated. Figure S4. (A) Quantitative host range analyses of 15 Synechococcus host strains against 138 cyanophage isolates testing the efficacy of infection. (B) Analysis of mean infectivity of coastal and upwelling phages in lineage I, II, IV, and VI reveal statistically different infectivity phenotypes at either site with T-test p <0.05 (*). Statistical significance was not assessed for lineages III and V due to low sample size nor on the original isolation host, WH7803 (†). Table S8. Corrected Rand Indices and Malia’s VI values to hierarchical clustering between the original host range matrix and a randomized host range matrix. The hierarchical clusters were split into different number of clusters (5,10, 20 and 50) for the analyses. The analyses revealed low correspondence between clustering, indicating that the clustering we observe in the original shared genes matrix is not random and that there is some correlation with a biological signal. Table S9. Fisher’s exact tests p-values and phi coefficients (for effect size) for genes found under positive selection in comparisons between phylogenomic lineages using the non-polarized McDonald-Kreitman test. Also, the table reflects the corresponding protein clusters in the GOV population dataset [19]. Table S10. Genic versus intergenic recombination breakpoints for each lineage. (DOC 250 kb) [file 12864_2016_3286_MOESM3_ESM.doc]

**Additional file 3: Testing genetic drift, selection, and recombination**

**Table S7.** F_ST_ values for genetic differentiation between coastal and offshore populations within each cyanophage lineage. The F_ST_ value for lineage II is most likely high due to the low number of representatives and lack of diversity among the offshore lineage II phages. Due to the low number of individuals isolated from the offshore site for some of the clusters, F_ST_ could not be calculated.

**Fig. S4. (A)** Quantitative host range analyses of 15 *Synechococcus* host strains against 138 cyanophage isolates testing the efficacy of infection. **(B)** Analysis of mean infectivity of coastal and upwelling phages in lineage I, II, IV, and VI reveal statistically different infectivity phenotypes at either site with T-test p <0.05 (*). Statistical significance was not assessed for lineages III and V due to low sample size nor on the original isolation host, WH7803 (†).

**Table S8.** Corrected Rand Indices and Malia’s VI values to hierarchical clustering between the original host range matrix and a randomized host range matrix. The hierarchical clusters were split into different number of clusters (5,10, 20 and 50) for the analyses. The analyses revealed low correspondence between clustering, indicating that the clustering we observe in the original shared genes matrix is not random and that there is some correlation with a biological signal.

**Table S9.** Fisher’s exact tests p-values and phi coefficients (for effect size) for genes found under positive selection in comparisons between phylogenomic lineages using the non-polarized McDonald-Kreitman test. Also, the table reflects the corresponding protein clusters in the GOV population dataset [19].

**Table S10.** Genic versus intergenic recombination breakpoints for each lineage.

**Table S7:**

| **Lineage** | **F_ST_** |
| --- | --- |
| I | 0.0097 |
| II | 0.3584 |
| III | N/A |
| IV | 0.0012 |
| V | N/A |
| VI | 0.0000 |

**Fig. S4:**

**Table S8:**

| **Number of clusters compared** | **Corrected Rand Index** | **Malia’s VI** |
| --- | --- | --- |
| 5 | -0.09988513 | 1.317719 |
| 10 | -0.07308659 | 2.606419 |
| 20 | -0.03138583 | 3.560547 |
| 50 | -0.002148682 | 2.89116 |

**Table S9:**

| **Lineage** | **Lineage Comparison** | **Protein Cluster** | **Corresponding GOV Protein Cluster** | **Annotation** | **P-value** | **Phi Correlation** |
| --- | --- | --- | --- | --- | --- | --- |
| I | II | MBARI_Cyanophage_PC_176 | PC_003712 | hypothetical protein | 0.00 | -0.20 |
| II | III | MBARI_Cyanophage_PC_82 | PC_000633 | hypothetical protein | 0.01 | -0.26 |
| II | I | MBARI_Cyanophage_PC_82 | PC_000633 | hypothetical protein | 0.00 | -0.16 |
| III | V | MBARI_Cyanophage_PC_105 | PC_007505 | PurL 5 5-phosphoribosylformylglycinamide amidotransferase | 0.02 | -0.33 |
| III | II | MBARI_Cyanophage_PC_105 | PC_007505 | PurL 5 5-phosphoribosylformylglycinamide amidotransferase | 0.01 | -0.29 |
| III | IV | MBARI_Cyanophage_PC_105 | PC_007505 | PurL 5 5-phosphoribosylformylglycinamide amidotransferase | 0.00 | -0.23 |
| III | I | MBARI_Cyanophage_PC_105 | PC_007505 | PurL 5 5-phosphoribosylformylglycinamide amidotransferase | 0.04 | -0.18 |
| III | V | MBARI_Cyanophage_PC_106 | PC_000834 | hypothetical protein | 0.00 | -0.50 |
| III | II | MBARI_Cyanophage_PC_106 | PC_000834 | hypothetical protein | 0.00 | -0.33 |
| III | I | MBARI_Cyanophage_PC_106 | PC_000834 | hypothetical protein | 0.00 | -0.33 |
| III | II | MBARI_Cyanophage_PC_137 | PC_004313 | hypothetical protein | 0.00 | -0.29 |
| III | I | MBARI_Cyanophage_PC_137 | PC_004313 | hypothetical protein | 0.00 | -0.28 |
| III | I | MBARI_Cyanophage_PC_175 | PC_003787 | hypothetical protein | 0.03 | -0.13 |
| III | I | MBARI_Cyanophage_PC_27 | PC_000302 | gp14 neck protein | 0.00 | -0.22 |
| III | II | MBARI_Cyanophage_PC_27 | PC_000302 | gp14 neck protein | 0.00 | -0.21 |
| III | II | MBARI_Cyanophage_PC_30 | PC_001821 | hypothetical protein | 0.00 | -0.37 |
| III | V | MBARI_Cyanophage_PC_44 | PC_001338 | DNA adenine methylase Dam | 0.01 | -0.12 |
| III | I | MBARI_Cyanophage_PC_90 | PC_005077 | hypothetical protein | 0.02 | -0.23 |
| IV | V | MBARI_Cyanophage_PC_42 | PC_000088 | hypothetical protein | 0.00 | -0.43 |
| IV | III | MBARI_Cyanophage_PC_42 | PC_000088 | hypothetical protein | 0.00 | -0.14 |
| IV | V | MBARI_Cyanophage_PC_62 | PC_003933 | hypothetical protein | 0.00 | -0.39 |
| IV | I | MBARI_Cyanophage_PC_62 | PC_003933 | hypothetical protein | 0.00 | -0.16 |
| IV | V | MBARI_Cyanophage_PC_77 | PC_003449 | hypothetical protein | 0.01 | -0.29 |
| IV | I | MBARI_Cyanophage_PC_77 | PC_003449 | hypothetical protein | 0.00 | -0.12 |
| VI | V | MBARI_Cyanophage_PC_55 | PC_000646 | PsbA photosystem II D1 protein | 0.00 | -0.13 |
| VI | I | MBARI_Cyanophage_PC_56 | PC_000646 | PsbD photosystem II D2 protein | 0.00 | -0.29 |
| VI | V | MBARI_Cyanophage_PC_56 | PC_000646 | PsbD photosystem II D2 protein | 0.00 | -0.27 |
| VI | III | MBARI_Cyanophage_PC_56 | PC_000646 | PsbD photosystem II D2 protein | 0.00 | -0.26 |
| VI | II | MBARI_Cyanophage_PC_56 | PC_000646 | PsbD photosystem II D2 protein | 0.00 | -0.20 |
| VI | V | MBARI_Cyanophage_PC_60 | PC_002409 /  PC_012106 | Hli03 high light inducible protein | 0.00 | -0.38 |
| VI | II | MBARI_Cyanophage_PC_60 | PC_002409 /  PC_012106 | Hli03 high light inducible protein | 0.00 | -0.14 |
| VI | I | MBARI_Cyanophage_PC_60 | PC_002409 /  PC_012106 | Hli03 high light inducible protein | 0.00 | -0.13 |
| VI | III | MBARI_Cyanophage_PC_60 | PC_002409 /  PC_012106 | Hli03 high light inducible protein | 0.00 | -0.13 |
